# CD44-mediated monocyte transmigration across *Cryptococcus neoformans-*infected brain microvascular endothelial cells is enhanced by HIV-1 gp41-I90 ectodomain

**DOI:** 10.1186/s12929-016-0247-2

**Published:** 2016-02-20

**Authors:** Xiaolong He, Xiaolu Shi, Santhosh Puthiyakunnon, Like Zhang, Qing Zeng, Yan Li, Swapna Boddu, Jiawen Qiu, Zhihao Lai, Chao Ma, Yulong Xie, Min Long, Lei Du, Sheng-He Huang, Hong Cao

**Affiliations:** Department of Microbiology, Guangdong Provincial Key Laboratory of Tropical Disease Research, School of Public Health and Tropical Medicine, Southern Medical University, Guangzhou, 510515 China; The Second School of Clinical Medicine, Southern Medical University, Guangzhou, 510515 China; Saban Research Institute, Children’s Hospital Los Angeles, University of Southern California, Los Angeles, CA 90027 USA

**Keywords:** *Cryptococcus neoformans*, HIV-1 gp41-I90 ectodomain, Blood-brain barrier, CD44

## Abstract

**Background:**

*Cryptococcus neoformans* (Cn) is an important opportunistic pathogen in the immunocompromised people, including AIDS patients, which leads to fatal cryptococcal meningitis with high mortality rate. Previous researches have shown that HIV-1 gp41-I90 ectodomain can enhance Cn adhesion to and invasion of brain microvascular endothelial cell (BMEC), which constitutes the blood brain barrier (BBB). However, little is known about the role of HIV-1 gp41-I90 in the monocyte transmigration across Cn-infected BBB. In the present study, we provide evidence that HIV-1 gp41-I90 and Cn synergistically enhance monocytes transmigration across the BBB in vitro and in vivo. The underlying mechanisms for this phenomenon require further study.

**Methods:**

In this study, the enhancing role of HIV-1 gp41-I90 in monocyte transmigration across Cn-infected BBB was demonstrated by performed transmigration assays in vitro and in vivo.

**Results:**

Our results showed that the transmigration rate of monocytes are positively associated with Cn and/or HIV-1 gp41-I90, the co-exposure (HIV-1 gp41-I90 + Cn) group showed a higher THP-1 transmigration rate (*P <* 0.01). Using CD44 knock-down HBMEC or CD44 inhibitor Bikunin in the assay, the facilitation of transmigration rates of monocyte enhanced by HIV-1 gp41-I90 was significantly suppressed. Western blotting analysis and biotin/avidin enzyme-linked immunosorbent assays (BA-ELISAs) showed that Cn and HIV-1 gp41-I90 could increase the expression of CD44 and ICAM-1 on the HBMEC. Moreover, Cn and/or HIV-1 gp41-I90 could also induce CD44 redistribution to the membrane lipid rafts. By establishing the mouse cryptococcal meningitis model, we found that HIV-1 gp41-I90 and Cn could synergistically enhance the monocytes transmigration, increase the BBB permeability and injury in vivo.

**Conclusions:**

Collectively, our findings suggested that HIV-1 gp41-I90 ectodomain can enhance the transmigration of THP-1 through Cn-infected BBB, which may be mediated by CD44. This novel study enlightens the future prospects to elaborate the inflammatory responses induced by HIV-1 gp41-I90 ectodomain and to effectively eliminate the opportunistic infections in AIDS patients.

## Background

*Cryptococcus neoformans* (Cn) is an important pathogenic fungus with capsule and causes severe meningitis and disseminated infections, especially in patients with defective cellular immunity, such as AIDS patients [1, 2]. Cryptococcosis is the most common opportunistic fungal infection and one of the major causes of death in AIDS patients (mortality rate ~ 30 %) [3, 4]. Despite major advances in the treatment of HIV-1 infection with Highly Active Antiretroviral Therapy (HAART), cryptococcosis remains prevalent even in developed countries [5–9]. Cn infects mainly through the respiratory tract, spreads from the pulmonary circulation to the brain tissues, resulting in meningitis [10, 11]. The pathogenesis of *cryptococcal* meningitis (CM) is still largely unknown, while it is well known that crossing the BBB is the pivotal step leading to the development of meningitis. The damage of the BBB is generally induced by the interactions between pathogens and brain microvascular endothelial cells (BMECs), which leads to edema and increased permeability, and subsequently facilitate more interactions between the immune cells and BMECs [12]. Previous research had shown that Cn is able to cause considerable morphological changes and actin reorganization in HBMEC [1]. Many signaling molecules, including CD44, caveolin-1, PKCα, endocytic kinase DYRK3, in lipid rafts have been characterized and shown to play an important role during the Cn internalization [2, 13–16].

Cryptococcosis is one of the most fatal co-morbidity factors of AIDS. The interrelationship between HIV-1 and Cn is intriguing and intricate, as both pathogens cause severe neuropathological complications. The details of how HIV-1 virotoxins, including gp120 and gp41, enhance Cn invasion of the BBB are still largely unknown. Our recent study has shown that HIV-1-gp41-I90 has a remarkable effect in promoting the adhesion and invasion of Cn [17]. Through construction of a recombinant protein, HIV-1 gp41-I90, which is the ectodomain of gp41 (amino acid residues 579–611), we have shown that HIV-1 gp41-I90 ectodomain could activate many molecular events including up-regulation of ICAM-1 on the HBMEC, redistribution of CD44 and β-actin on the lipid rafts and induction of membrane ruffling on the surface of HBMEC. These events could enhance brain invasion by Cn and eventually can lead to severe HIV-1-associated CM [17, 18]. CD44 is a cell-surface glycoprotein involved in cell–cell interactions, cell adhesion and migration, which is widely distributed in a variety of endothelial cells, including HBMEC [19]. The interaction between hyaluronic acid (HA) on the Cn and its receptor CD44 on the surface of HBMEC is the initial step in cryptococcal brain invasion [13]. The role played by CD44/HA in the interaction between BMECs and leukocytes and the exudation of leukocyte is previously characterized [13]. CD44 has also been proposed to play an important role in Cn infection-induced adhesion and transmigration activities of leukocyte. It is reasonable to speculate that CD44 could also be important for HIV-1 gp41-I90 ectodomain mediated brain invasion of Cn.

Delineating the mechanism of Cn transmigration across the BBB is essential to explore the potential of HIV-1 in enhancing the brain invasion by Cn. Many research groups have suggested three possible routes of Cn transmigration across the BBB: (1) Trans-cellular passage through endothelial cells by a specific ligand-receptor interaction [1, 20], this mode of invasion has been observed for *Escherichia coli* [21–23], group B *Streptococcus* [24], *Listeria monocytogenes* [25], *Neisseria meningitides* [26] and the fungal pathogen *Candida albicans* [27]; (2) Paracellular penetration after mechanical or biochemical disruption of the BBB [1, 28, 29], just like the protozoan *Trypanosomasp* [30, 31]; (3)“Trojan horse” method, in which the infected immune cells, such as monocytes carry the pathogen through the BBB, a method of infection by HIV-1 and simian immunodeficiency virus [32–34]. The existence of a Trojan horse method of crossing the BBB by Cn has been proved in a study by Caroline Charlier et al. [35]. Through infecting bone marrow-derived monocytes (BMDM) with Cn in vitro, the authors showed that fungal loads in brain of mice treated with Cn-infected BMDM were much higher than the control group. Accumulating evidence shows that Cn can use multiple means of transmigration and disruption of the BBB.

Previous research had shown that HIV-1 infection is able to increase the monocyte capacity to migrate across the BBB [36]. As existence of a Trojan horse method of crossing the BBB by Cn, it is reasonable to speculate that HIV-1 enhanced transmigration activity of monocytes might be responsible for severe brain disorder caused by Cn. In present study, through performing transmigration assays, we found that Cn and HIV-1 gp41-I90 could synergistically enhance monocytes transmigration across the BBB. Our findings provide a new idea for understanding the interrelationship between HIV-1 and Cn during the pathogenic progress of HIV-1-associated CM.

## Methods

### Chemicals and reagent

Evans blue (EB), L-(−)-Fucose, biotinylation kit and Caveolae/Rafts Isolation kit were purchased from Sigma-Aldrich (St. Louis, MO). Dynabeads M-450 Tosylactivated was purchased from Invitrogen (Carlsbad, CA). Ulex europaeus I (UEA I) lectin and mounting medium with DAPI were purchased from Vector (Buringame, CA). The HIV-1 gp41-I90 ectodomain peptide (gp41-I90) was prepared as previously described [37]. Recombinant HIV-1 Tat clade B protein and HIV-1 p24 recombinant were purchased from Prospec (Rehovort, Israel). All primary antibodies (Ab) were purchased from the commercial sources: a rabbit anti-MSFD2 Ab (sc-135305), a rabbit anti-CD44 Ab, a rat anti-Ly6C Ab and a rabbit anti-ICAM-1 Ab (Abcam, USA), a PE-conjugated anti-CD146 Ab (12-1469-41) and a PE-conjugated rat anti-mouse Ly6C Ab from eBiosciences (San Diego, CA, USA). The rest chemicals were obtained from Ding Guo Chang Sheng Company, Beijing, China.

### Fungi strains, cell lines and cultures

Cn wild strains B-4500FO2 was a generous gift from A Jong (University of Southern California, Los Angeles, USA). Yeast cells were grown aerobically at 30 °C in 1 % yeast extract, 2 % peptone and 2 % dextrose (YPD broth). Cells were harvested at early log phase, washed with phosphate-buffered saline (PBS) and resuspended. The yeast cell number was determined by direct counting from a hemocytometer [17]. Heat-inactivated Cn (H-Cn) was obtained by heating the microorganisms three times at 121 °C for 15 min [38]. Only batches that showed no re-growth in YPD broth were employed. HBMEC were isolated and cultured as described previously [39–41], which were grown in RPMI 1640 medium supplemented with 10 % heat-inactivated fetal bovine serum, 10 % Nu-serum, 2 mM glutamine, 1 mM sodium pyruvate, nonessential amino acids, vitamins, penicillin G (50 μg/ml) and streptomycin (100 μg/ml) at 37 °C in 5 % CO2. Cells were detached by trypsin-EDTA and subcultured on collagen-coated Transwell (3 μm pore size, 6.5-mm diameter) (BD Biosciences, San Jose, CA, USA) from T-25 flasks when ~70 %–80 % confluent. HBMEC monolayers on Transwell filters were monitored by measuring trans-endothelial electrical resistance (TEER) changes across the endothelial cell monolayer using an End Ohm epithelial voltohmeter (World Precision Instruments, Sarasota, FL, USA) [1, 27]. The cells are positive for factor VIII and fluorescently labeled acetylated low-density lipoprotein (Dil-AcLDL) uptake, demonstrating their endothelial origin and also express gamma glutamyl transpeptidase (GGT) and carbonic anhydrase (CA) IV, indicating their brain origin [42]. HBMEC are polarized and exhibit an average TEER value of 250–300Ω/cm^2^ [1]. The cells also exhibit the typical characteristics for brain endothelial cells expressing tight junctions and maintaining apical-to-basal polarity. THP-1 cells were purchased from the cell bank of Chinese Academy of Sciences and grown in RPMI 1640 medium supplemented with 10 % heat-inactivated fetal bovine serum, penicillin G (50 μg/ml) and streptomycin (100 μg/ml) at 37 °C in 5 % CO_2_.

### Mice

The C57BL/6 background mice (6 weeks of age) were brought from Animal Experimental Center of Southern Medical University (Guangzhou, China) and kept in the animal facility. They were raised in plastic cages and given food and water ad libitum. All experiments were approved by the ethics committee of Southern Medical University.

### CRISPR/Cas9-Mediated knockdown-CD44

The CRISPR-Cas9 system was used in our study to mediate down-regulated expression of CD44 in HBMEC. Human CD44 cDNA sequence was obtained from Gen Bank (NM_000610) and two pairs of single guide RNA (sgRNA) sequences (named CD44-1 and CD44-2, as below) were designed online (http://www.e-crisp.org/E-CRISP/designcrispr.html). The underlined sequences targeted the CD44 gene, and the bold italic letters indicate the *BsmBI* site. A 20 bp scrambled sequence (see below) was defined as a scramble control which was marked with “SC” in the text.

sgCD44-1:F:5’-***CACCG***CTACAGCATCTCTCGGACGG-3’,

R:3’-***C***GATGTCGTAGAGAGCCTGCC***CAAA*-**5’

sgCD44-2:F:5’-***CACCG***GGCACTCACCGATCTGCGCC-3’,

R: 3’-***C***CCGTGAGTGGCTAGACGCGG***CAAA*-**5’

Scramble Control:F:5’-***CACCG***GCACTACCAGAGCTAACTCA-3’,

R:3’-***C***CGTGATGGTCTCGATTGAGT***CAAA*-**5’

These sequences were annealed in 10 × T4 Ligation Buffer (NEB) withT4 PNK (NEB M0201S) by incubating oligonucleotides for 30 min at 37 °C, 5 min at 95 °C and ramping down to 25 °C at 5 °C/min, followed by slow cooling to 4 °C. The annealed DNA fragments were ligated into *BsmBI* sites of lentiCRISPRv2 (provided by Bao Zhang, Southern Medical University) to generate lentiCRISPRv2-CD44-1, lentiCRISPRv2-CD44-2 and lentiCRISPRv2-SC plasmids, respectively. These plasmids were transfected into 293 T cells with lentiviral packaging vectors pCMV-dR8.2 dvpr and pCMV-VSV-G (both provided by Bao Zhang, Southern Medical University) using lipofectamine 2000. Viruses were collected from the media 48 h post-transfection. HBMEC grown on 24 well plates were infected with collected viruses for 24 h in the presence of polybrene (Santa Cruz). Stably transfected clones were picked and maintained in medium containing 2 μg/ml puromycin for additional studies. Expression level of CD44 in stable cell line was analyzed by western blotting using anti-CD44 monoclonal antibodies. We assigned the stable cell line as KD-CD44 HBMEC in our study.

### THP-1 adhesion assay

THP-1 adhesion assays were performed as described by Che et al. [43]. Briefly, confluent HBMEC monolayers on 24-well plates were stimulated with different concentrations of Cn (10^5^-2 × 10^7^ CFU/ml) or gp41-I90 (0.02–20 μM) for 6 h. For the time-course study, confluent HBMEC monolayers were stimulated at different time intervals (0–24 h) with a single dose of Cn (5 × 10^6^ CFU/ml) or HIV-1 gp41-I90 (2 μM). After the incubation, monolayers were washed with PBS for four times. Each well was added with 1 × 10^6^ THP-1 and incubated with 90 min at 37 °C. Then, cells were washed for 5 times and fixed with 4 % paraformaldehyde in PBS. Assays were performed in triplicate wells. Fifteen microscope fields were randomly selected from three wells for each treatment to count the number of adherent monocytes and the data were analyzed using analysis of variance (ANOVA).

### THP-1 transmigration assay

THP-1 transmigration assays were performed as described previously [44, 45] with modification. HBMECs or KD-CD44 HBMECs were cultured in trans-well filters (3 μm pore size, 6 mm diameter, Millipore). In order to exclude the possibility that the monocytes migration elicited was due to destruction of HBMEC, the integrity of the monolayer was inspected by TEER and microscopy before the start of the assay. For HBMEC stimulation, different doses of Cn or HIV-1 gp41-I90 were added to the upper chambers with 0.8 ml EM (EM; containing 49 % M199, 49 % Ham’s F12, 1 mM sodium pyruvate and 2 mM L-glutamine) for 6 h. For the time-course study, HBMEC were stimulated at different time intervals (0–24 h) with a single dose of Cn (5 × 10^6^ CFU/ml) Cn or HIV-1 gp41-I90 (2 μM). After stimulation, THP-1 (1 × 10^6^ cells in 0.2 ml of EM) were added to the upper chamber and allowed to migrate over for 4 h (Dose response and kinetic assays were performed in advance to determine the optimized concentration and migration duration). At the end of the incubation, migrated THP-1 cells were collected from the lower chamber and counted in a blinded-fashion using a hemacytometer [43]. Final results of THP-1 transmigration were expressed as the percentage of THP-1 across the BMEC monolayers. For Bikunin treatment, BMEC were incubated with Bikunin (Gen-Script Corp., catalog no. 300233) in both upper and lower chambers for 1 h before stimulation [16]. The pre-treating time of bikunin was determined according to kinetic assays. The Bikunin was present throughout the monocytes transmigration experiment until the end.

### Assays of surface expression of CD44 and ICAM-1

As ICAM-1 and CD44 play a role in the leukocyte transmigration process during inflammatory, we next performed BA-ELISAs to measured the expression of CD44 and ICAM-1 on HBMEC. Before the assays, ICAM-1 and CD44 antibody were biotinylated with biotin using a biotinylation kit as described by the manufacturer. The methods for ELISAs were similar to those described previously [43]. HBMEC monolayers which grown on Transwell were treated with Cn (5 × 10^6^ CFU/ml) and HIV-1 gp41-I90 (2 μM) alone or joint use of them and incubated for 6 h. Treated monolayers were washed three times with PBS, fixed with 4 % paraformaldehyde and blocked for 30 min with PBS containing 5 % BSA. Biotin conjugated ICAM-1 antibody or CD44 antibody were added immediately after the blocking step. Incubation was carried out for 1 h at 37 °C. Cells were washed five times with PBS added 1 % BSA and incubated with peroxidase-conjugated avidin for 45 min at 37 °C. After the avidin incubation, cells were washed five times and liquid TMB substrate was added. The liquid was transferred to an ELISA plate after 15 min. Equal volume stop solution was added, and optical density at 450 nm was read. For each ELISA, an isotype-matched control antibody was used in place of the primary antibody in three wells, and this background was subtracted from the signal.

### Preparation of membrane lipid rafts from HBMECs

Lipid rafts were extracted using Caveolae/Rafts Isolation kit as described previously [13]. For each sample, HBMECs were grown in a 6 well plates for 2 days. On the day of the experiment, the cells were individually incubated with either PBS (control), or 2 μM HIV-1 gp41-I90, or 5 × 10^6^ CFU/ml Cn or 5 × 10^6^ CFU/ml Cn + 2 μM HIV-1 gp41-I90 individually for 6 h in the experimental medium. After incubation, the cells were washed with PBS three times, scraped in PBS and spun down at 750 g at 4 °C. Cell pellets were lysed in 200 μl of TN solution [25 mM Tris/HCl (pH 7.5), 1 mM DTT (dithiothreitol), a cocktail of protease inhibitors, 10 % sucrose and 1 % Triton X-100] on ice, and incubated for 30 min on ice. Samples were mixed with 1.16 ml of ice-cold OptiPrep^TM^, transferred into SW40 centrifuge tubes and overlaid with 2 ml each of 30, 30, 25, 20 and 0 % OptiPrep^TM^ in TN buffer. The gradients were spun at 35000 r.p.m. in an SW40 rotor for 5 h at 4 °C. Nine fractions were collected from the top to the bottom of centrifuge tubes. For western blotting, equal amounts of proteins from each fraction were used. Rabbit anti-CD44 Ab (Abcam, 1:5000 dilution) and anti-rabbit-HRP conjugate (1:500 dilution) were used in these experiments.

### Western blotting analysis

To assess Cn or HIV-1 gp41-I90 induced expression of CD44 and ICAM-1 on HBMEC, monolayers was subjected to individual treatment with PBS, 5 × 10^6^ CFU/ml Cn, 2 μM HIV-1 gp41-I90, 5 × 10^6^ CFU/ml Cn + 2 μM HIV-1 gp41-I90 or 0, 0.2, 2 and 20 μM HIV-1 gp41-I90 respectively for 6 h at 37 °C in 5 % CO_2_. After incubation, the cells were collected and lysed on ice in lysis buffer [1 × PBS, 1 % NP40, 0.1 % sodium dodecyl sulphate, 5 mm ethylenediaminetetraacetic acid (EDTA), 0.5 % sodium deoxycholate, 1 mm sodium orthovanadate] with protease inhibitors. The protein concentration was measured using the Bradford protein assay (Beyotime Institute of Biotechnology, Shanghai, China). Equal amounts of proteins were separated electrophoretically and transferred onto polyvinylidene difluoride membranes (Millipore). Each membrane was probed with a rabbit anti-CD44 antibody (1:5000) or rabbit anti-ICAM-1antibody (1:200). Expression of protein was examined with a horseradish peroxidase-conjugated anti-rabbit IgG and enhanced chemiluminescence (Pierce, Rockford, IL, USA). A goat polyclonal anti-β-actin antibody (1:1500; Santa Cruz Biotechnology) was used to confirm equal loading of proteins. The intensity of the bands was scanned and analyzed with Alpha Imager gel documentation system and analysis software.

### Mouse cryptococcal meningitis model

All the animal experiments were performed strictly according to the guidelines for animal care in Southern Medical University (China). Our protocols were approved (Approval No. 2014A016) by the School of Public Health and Tropical Medicine of Southern Medical University, which obtained the permission for performing the research protocols and all animal experiments conducted during the present study from the ethics committee of Southern Medical University. All surgery was performed under anesthesia with ketamine and lidocaine, and all efforts were made to minimize suffering. For study the role of HIV-1 gp41-I90 on Cn-caused monocyte recruitment into the CNS of mice, mouse cryptococcal meningitis model was established as described previously [17]. 6 weeks-old C57BL/6 mice (6 mice each group) were intravenously injected with 10^6^ Cn cells via the tail vein, with or without HIV-1 gp41-I90 (10 μg/g mouse weight). After 24 h injection, mice were anaesthetized with ketamine and lidocaine, and blood samples were collected from heart puncture for isolation and purification of mouse brain microvascular endothelial cells. After perfusion from heart puncture with 20 ml PBS, the skull was opened. CSF samples were collected by washing the brain tissues with 100 μl of PBS, and then by washing the cerebral ventricles and cranial cavity with another 100 μl of PBS. CSF samples containing more than 10 erythrocytes per μl were discarded as contaminated samples. As the expression level of CD14 is very low in mouse monocytes, anti-Ly6C Ab was used to determine monocyte in CSF [46]. Monocytes were stained with a PE-conjugated rat anti-mouse Ly6C Ab (eBiosciences, CA, USA) and counted under the fluorescence microscope.

### Isolation and purification of mouse brain microvascular endothelial cells

Recently, we have demonstrated that circulating BMECs (cBMECs) can be used as potential novel cell-based biomarkers for indexing of the BBB injury [47]. This technology was used by us to explore whether HIV-1 gp41-I90 is able to increase Cn-associated BBB damages in our study. Briefly, beads were prepared according to the manufacturer’s instructions (Invitrogen) and resuspended in Hanks’ balanced salt solution (HBSS, Invitrogen Corp., Carlsbad, CA, USA) plus 5 % fetal calf serum (HBSS + 5 %FCS) to a final concentration of 4 × l0^8^ beads/ml. The cBMECs were prepared as described previously [47, 48]. Endothelial cells from blood samples were isolated by absorption to Ulex-coated beads [49] and detached from the beads by fucose. Detached endothelial cells were adhered again to MFSD2a-coated beads. To counting the cBMECs from blood samples, cells adhered to MFSD2a-coated beads were labeled with PE-conjugated CD146 antibody and transferred to glass splices by cytospin for counting under a fluorescence microscope. These endothelial cells were positive for CD146 [47], demonstrating their endothelial origin, and also expressed MFSD2a [50], indicating their brain origin. Total cBMECs were identified based on their CD146 (endothelial cell marker)^+^/DAPI (nuclei)^+^phenotypes.

### Histopathology and immunohistochemistry

Mouse brain tissue was fixed in 4 % phosphate-buffered paraformaldehyde and was paraffin-embedded. Immunohistochemistry was performed on 5 μm paraffin tissue sections. Mouse monocytes were identified with anti-Ly6C (1:100; Abcam). To detect primary Abs, a goat anti-rabbit antibody conjugated with horseradish peroxidase was used with 50 mM Tris · HCl buffer (pH 7.4) containing DAB and H_2_O_2_, and the sections were lightly counterstained with hemotoxylin.

### Statistical analysis

Data are shown in mean ± standard deviation and analyzed by one-way ANOVA tests. All statistical analysis was carried out at 5 % level of significance and P value less than 0.05 was considered to be significant. SPSS software (version 13.0) was used for statistical analysis. The synergistic enhancing effect on joint use of Cn and HIV-1 gp41-I90 was analyzed using the CalcuSyn Software (Biosoft).

## Results

### Effect of Cn and HIV-1 gp41-I90 on adhesion and transmigration of THP-1

**Fig. 1:**
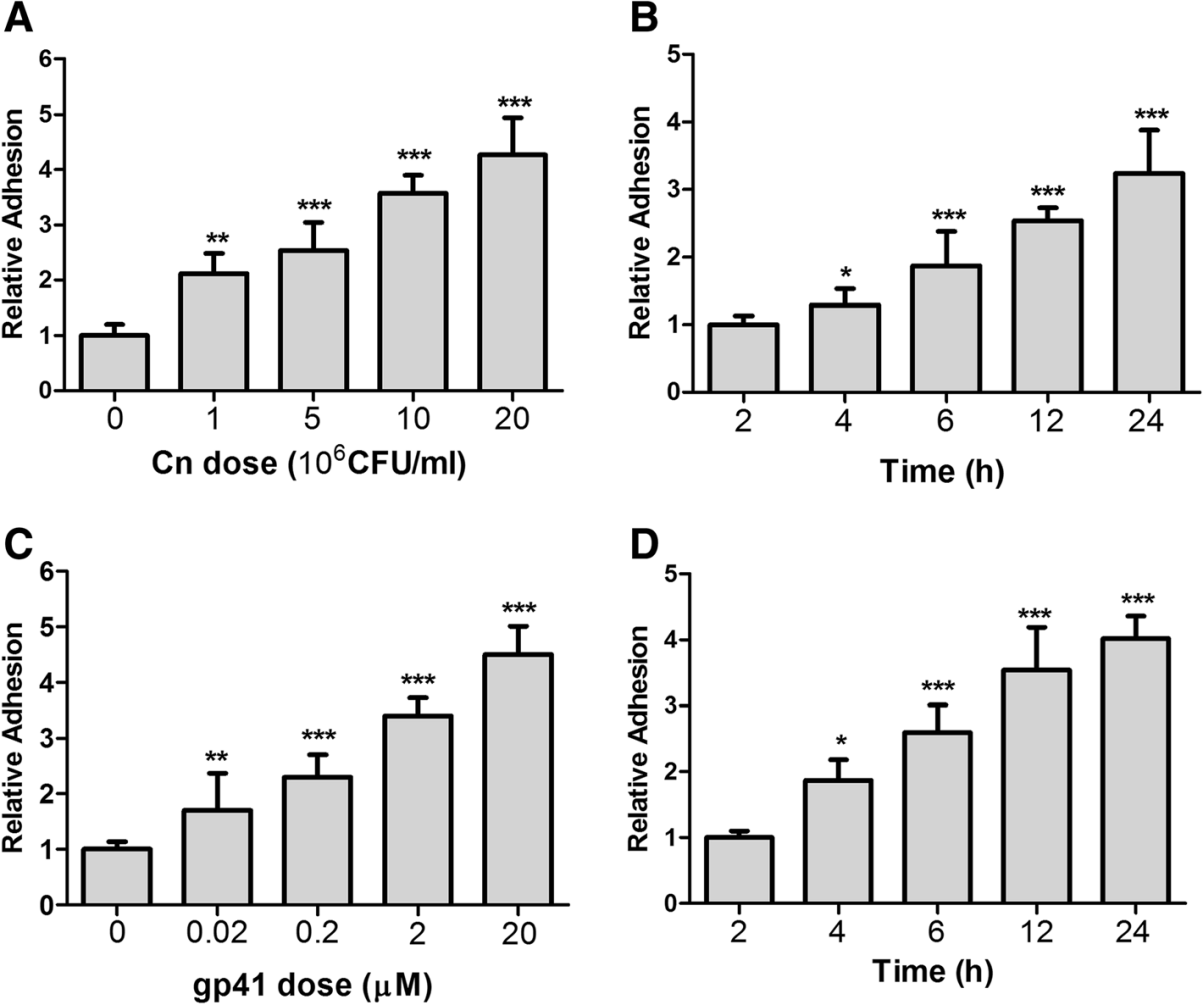
Cn and HIV-1 gp41-I90 induce THP-1 adhesion to HBMEC monolayers in a dose- and time-dependent manner. THP-1 adhesion assays were performed as described in the Methods section. **a**, **c** Induction of THP-1 adhesion with different doses (10^6^-2 × 10^7^ CFU/ml) of Cn and HIV-1 gp41 (0–20 μM). **b**, **d** Time-course study of Cn and HIV-1 gp41-I90-induced THP-1 adhesion to HBMEC monolayers. THP-1 adhesion was triggered by 5 × 10^6^ CFU/ml of Cn or 2 μM HIV-1 gp41-I90. Adhesion was expressed as an n-fold increase relative to the basal level. *P < 0.05, **P <0.01, ***P < 0.001

**Fig. 2:**
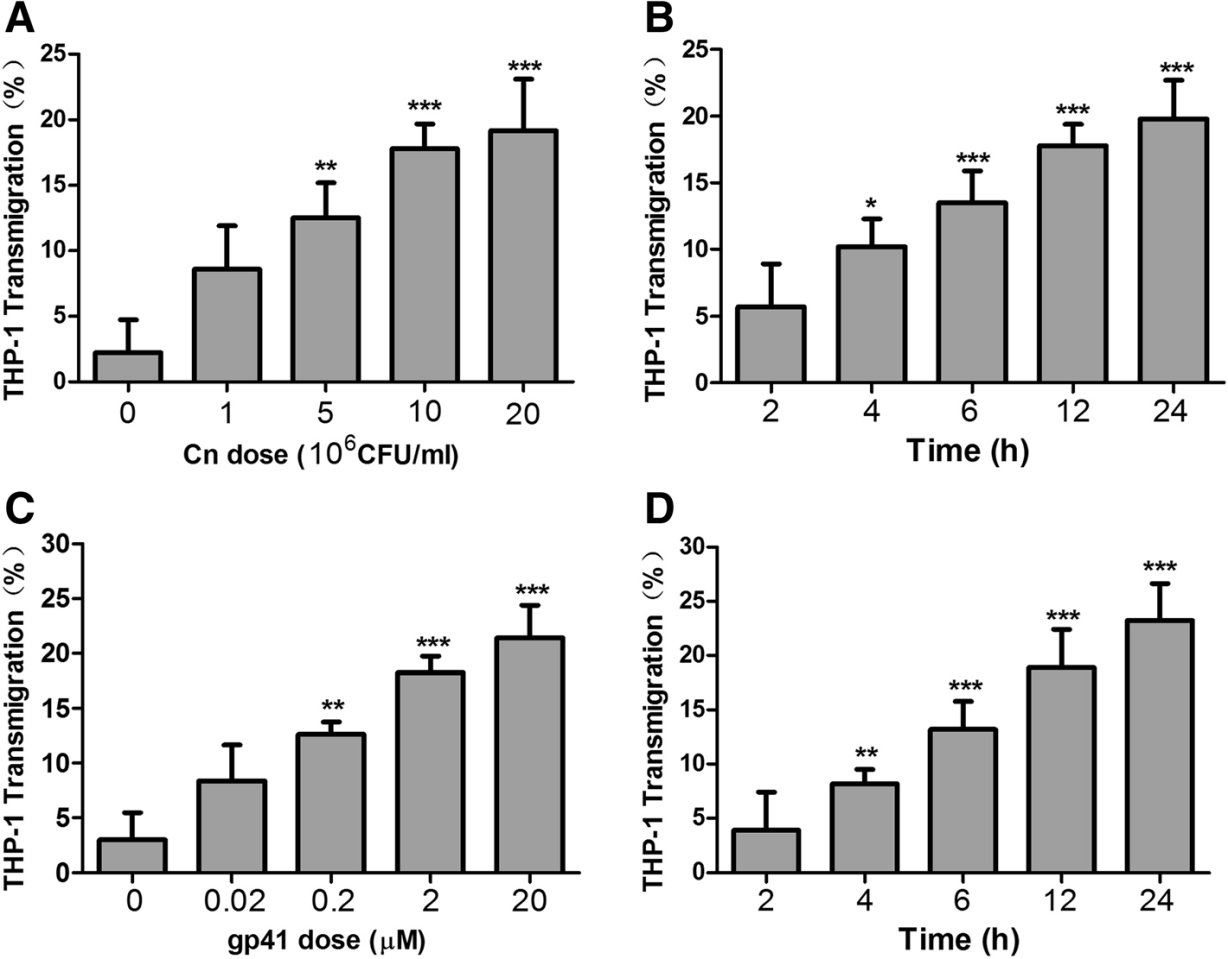
Cn and HIV-1 gp41-I90 induce THP-1 transmigration across HBMEC monolayers in a dose- and time-dependent manner. **a**, **c** Different doses (10^6^-2 × 10^7^ CFU/ml) of Cn and HIV-1 gp41-I90 (0–20 μM) induced THP-1 transmigration. **b**, **d** Time-course study of Cn and HIV-1 gp41-I90-induced THP-1 transmigration across HBMEC monolayers. THP-1 transmigration was triggered by 5 × 10^6^ CFU/ml of Cn or 2 μM HIV-1 gp41-I90. The values represent the mean percent transmigrating THP-1 of triplicate samples and are representative of one experiment from three independent experiments showing similar data. *P < 0.05, **P <0.01, ***P < 0.001 compared with control

Recruitment of monocytes into CNS plays an important role in the inflammatory response induced by fungal factors [51]. To determine the role of Cn and HIV-1 gp41-I90 on transmigration of monocytes, we first evaluated the effect of Cn and HIV-1 gp41-I90 on monocytes adhesion to HBMEC at different yeast doses (10^6^-2 × 10^7^ CFU/ml) and time intervals (0–24 h). Individually, as shown in Fig. 1a–d, Cn and gp41-I90 not only could dose-dependently induce adhesion of monocytes to HBMEC, but also it is time-dependent. Next, we performed transmigration assays to test whether Cn and HIV-1 gp41-I90 could induce monocytes transmigration across the BBB in vitro at a manner similar to adhesion. As we expected, Cn and HIV-1 gp41-I90 could also induce monocytes transmigration across the BBB in vitro in a dose- and time-dependent manner (Fig. 2a–d). In order to exclude the possibility that the increased transendothelial migration by Cn or HIV-1 gp41-I90 was due to disruption of the BBB, the integrity of the monolayer was inspected by determining the TEER across the monolayer. As shown in Fig. 5c, the TEER only declined to <8 % of the starting value after incubation with indicated doses of Cn and HIV-1 gp41-I90 or joint use of them. These results suggest that Cn and gp41-I90 could induce monocyte adhesion to and transmigration across the HBMEC monolayers.

### HIV-1 gp41-I90 and Cn synergistically enhance the adhesion and transmigration activity of monocytes

**Fig. 3:**
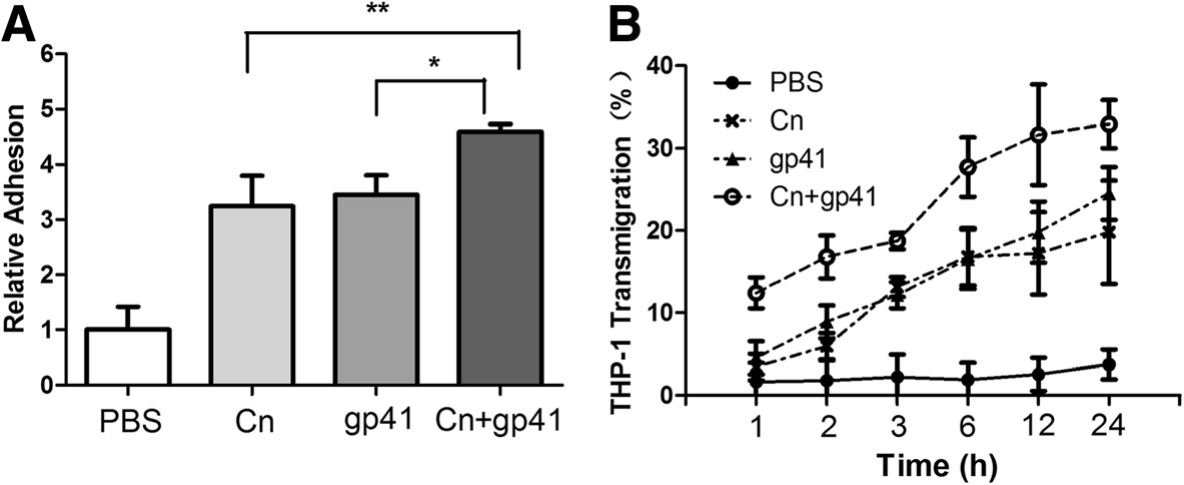
Cn and HIV-1 gp41-I90 co-induced adhesion and transmigration of THP-1 to HBMEC. **a** Induction of THP-1 adhesion with Cn and HIV-1 gp41 alone or Cn in combination with HIV-1 gp41. The adhesion of THP-1 was triggered by 5 × 10^6^ CFU/ml of Cn, 2 μM of HIV-1 gp41-I90 or both of them. Joint use of Cn and HIV-1 gp41-I90 significantly induced THP-1 adhesion at 1.5 h after addition of THP-1 to the monolayer when compared with use Cn and HIV-1 gp41 alone. **b** Time-course study of Cn and HIV-1 gp41-I90 induced THP-1 transmigration across HBMEC monolayers. THP-1 transmigration was triggered by 5 × 10^6^ CFU/ml Cn, 2 μM HIV-1 gp41-I90 or both of them. The values represent the mean of triplicate samples and are representative of one experiment from three independent experiments showing similar data. (*P < 0.05, **P < 0.01)

In this assay, the adhesion rate of THP-1 was measured in four groups: PBS, Cn, HIV-1 gp41-I90 and joint use of Cn and HIV-1 gp41-I90 (Fig. 3a). Compared with the control group, all other groups showed significant increase in the adhesion rates, among which the HIV-1 gp41-I90 + Cn group was the highest. For the time-course study of THP-1 transmigration, as shown in Fig. 3b, when the incubation time was increased to 24 h, the transmigration rate of HIV-1 gp41-I90 + Cn group increased to 32 % compared to the Cn group (19.2 %) and HIV-1 gp41-I90 group (21.8 %). Therefore, we concluded that the co-exposure of HIV-1 gp41-I90 and Cn in HBMEC has a significant time effect in transmigration of THP-1 cells. Moreover, the co-exposure group showed a higher rate initially, and the pro-migration effect was more durable as well. Determination of a synergistic effect of Cn and HIV-1 gp41-I90 combination was performed according to the median effect principle using the CalcuSyn Software (Biosoft) as described previously [52]. The CI values for the combination treatment of Cn and HIV-1 gp41-I90 were less than 1, suggesting that the combination is highly synergistic. These results suggested that HIV-1 gp41-I90 and Cn was able to synergistically enhance the adhesion and transmigration activity of monocytes.

### Specificity of synergistically enhanced transmigration activity of monocyte by Cn and HIV-1 gp41

**Fig. 4:**
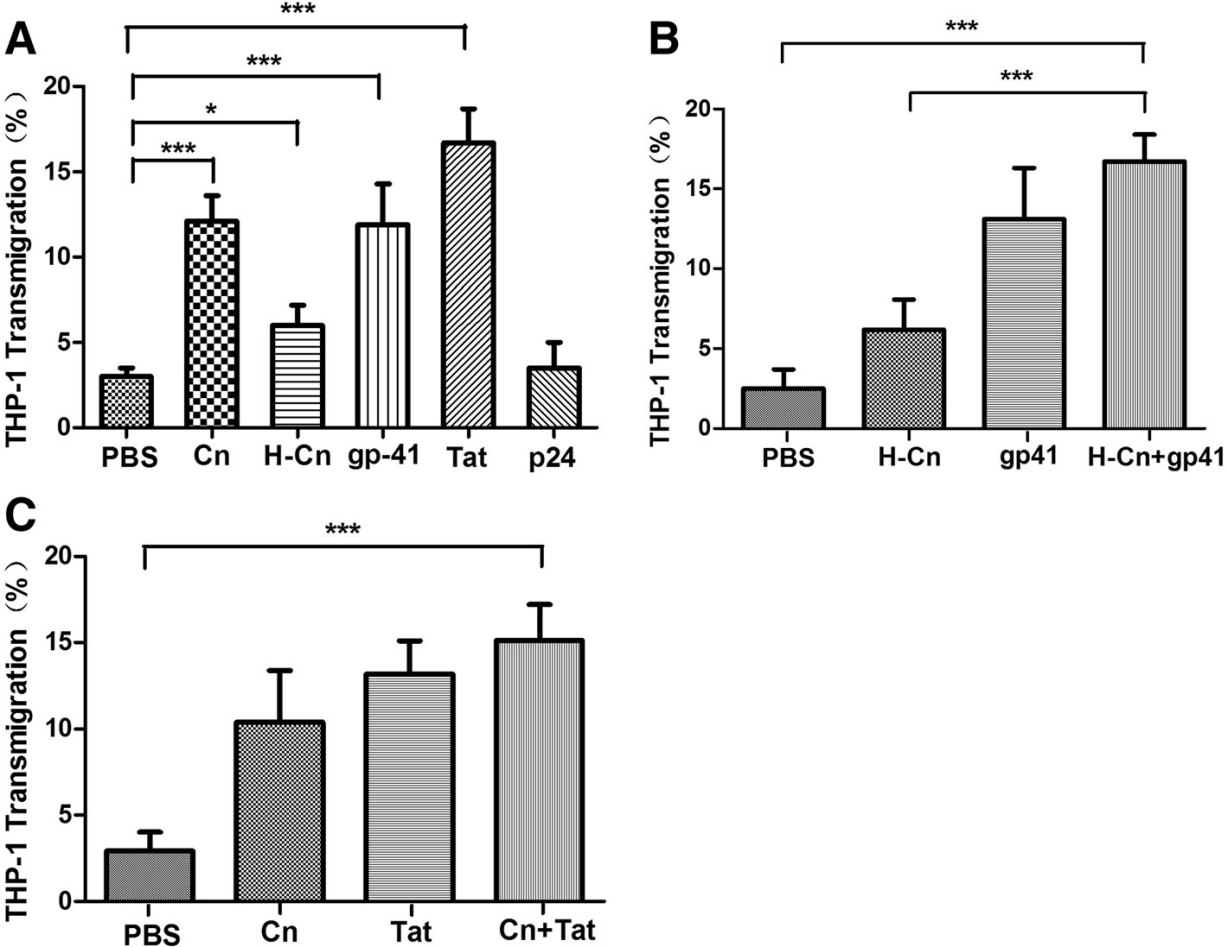
Specificity of Cn and HIV gp41 in induced monocyte transmigration across HBMEC monolayers. **a** Monocytes transmigration rate of Cn, H-Cn, HIV-1 gp41, HIV Tat, and HIV p24. HBMEC monolayers were treated with Cn, H-Cn, gp41, Tat or p24 respectively. Transmigration assays we performed as described as Methods section. **b** These are no synergistic effect of monocytes transmigration following BMEC treatment with H-Cn and HIV-1 gp41. **c** These are no synergistic effect of monocytes transmigration following BMEC treatment with Cn and HIV Tat. The values represent the mean percent transmigrating THP-1 of triplicate samples and are representative of one experiment from three independent experiments showing similar data. *P < 0.05, ***P < 0.001 compared with control

In Fig. 2, we showed evidence of a dose- and time-dependent increase in monocyte transmigration activity following BMEC treatment with Cn and HIV-1 gp41. However, it is not clear whether these enhancing effects are specific to Cn and gp41. In order to further investigate this issue, heat-inactivated Cn, HIV Tat and p24 were used in transmigration assays. Briefly, HBMECs cultured in trans-well filters were treated with either PBS (control), Cn (1 × 10^6^ CFU/ml), H-Cn (1 × 10^6^ CFU/ml), HIV-1 gp41 (0.2 μM), HIV Tat (0.2 μM) or HIV p24 (0.2 μM) for 6 h. THP-1 transmigration assays were performed as described as Methods section. Like Cn and HIV-1 gp41, as shown in Fig. 4a, H-Cn and HIV Tat could also increase monocytes transmigration across BBB. Among these stimulations, the HIV Tat molecule contributes a higher enhancement of monocytes transmigration across to BBB. Next, we performed transmigration assays again to further examine whether H-Cn and HIV-1 gp41 or Cn and HIV Tat could also synergistically enhance the transmigration activity of monocyte. However, as shown in Fig. 4a–c, none of them could synergistically enhance the transmigration activity of monocyte. The synergistic effect was determined using the CalcuSyn Software as described above.

### The enhancement of Cn and HIV-1 gp41-I90 in transmigration of monocytes across the BBB is closely related to CD44

**Fig. 5:**
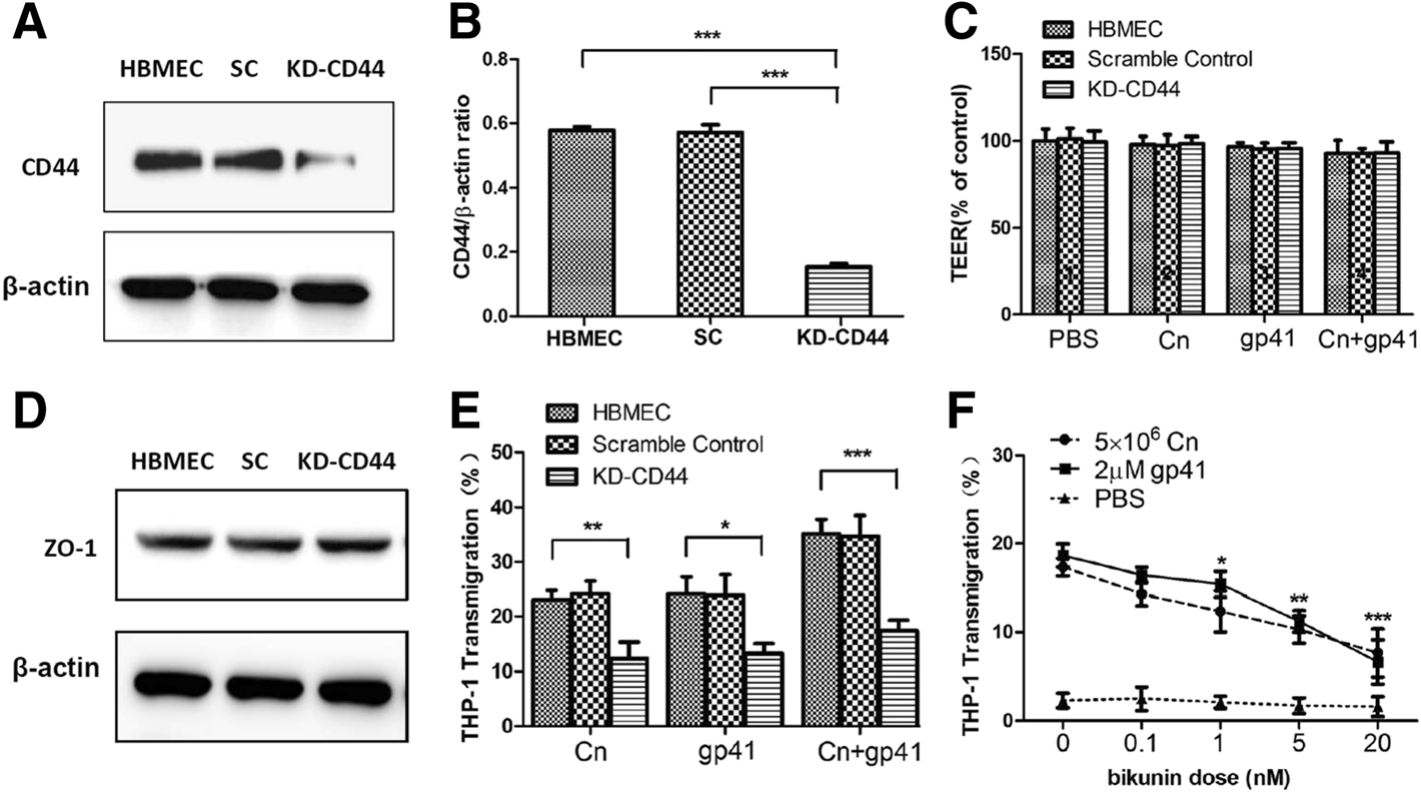
Genetic (Knock-down) and chemical (inhibitor) blockage of CD44 results in a reduction of THP-1 transmigration across HBMEC. **a**, **b** Down-regulating effect of the CRISPR Cas9 system was measured by Western blotting. β-actin was used as a loading control for each sample. Results showed a significant decrease (77 %) in the CD44/β-actin optical density ratio (***P <0.001). **c** Effect of Cn and HIV-1 gp41 on the BBB permeability, as evaluated by TEER. HBMEC, SC HBMEC or KD-CD44 HBMEC cultures were treated with Cn (2 × 10^7^ CFU/ml), HIV-1 gp41 (20 μm) alone or Cn in combination with HIV-1 gp41 for 6 h. Either Cn or HIV-1 gp41 alone or joint use of them had no significant effect on the permeability of HBMEC, SC HBMEC and KD-CD44. **d** Effect of down-regulated CD44 expression on ZO-1 in HBMEC. **e** Transmigration assays were performed with HBMEC, SC HBMEC and KD-CD44, a significant suppression of transmigration was observed in the KD-CD44 group. **f** Bikunin inhibits Cn- and HIV-1 gp41-I90- induced THP-1 transmigration across HBMEC in a dose-dependent manner. An uninfected BMEC as a negative control was designed in the assay. Results are expressed as the mean and standard deviation of quadruplicate assays. (*P < 0.05, **P <0.01, ***P < 0.001)

The HIV-1 envelope glycoprotein gp41 could up-regulate CD44 in AIDS patients with CM, which ultimately enhances the adhesion and invasion of Cn to BMECs [18, 53]. In order to examine whether Cn and HIV-1 gp41-I90 enhance the transmigration of monocytes across the BBB is mediated by CD44, two different blockage approaches, genetic knockdown (KD-CD44 HBMEC) and chemical inhibition (CD44 inhibitor Bikunin) were used. KD-CD44 HBMEC was generated by the CRISPR Cas 9 genome editing technique, which is an effective way to down-regulate expression of protein in a broad variety of mammalian cells [54, 55]. The down-regulating effect of Cas9 was measured at the protein level by Western blotting, approximately 77 % knock-down was achieved (Fig. 5a, b). In order to ensure that the barrier remains intact in the absence of CD44, the integrity of the barrier of KD-CD44 HBMEC was evaluated by TEER. As shown in Fig. 5c, stimulation of Cn and HIV-1 gp41-I90 alone or together has no significant effect on integrity of the barrier. Furthermore, we also performed Western blotting to examine the effect of down-regulated CD44 expression on tight junction protein ZO-1. As shown in Fig. 5d, the absence of CD44 has no effect on ZO-1 expression in HBMEC. THP-1 transmigration assays were performed with HBMEC, SC (scramble control) HBMEC and KD-CD44 HBMEC as described as Method section. As shown in Fig. 5e, significant reduction of THP-1 transmigration was observed in the KD-CD44 HBMEC groups.

Bikunin is a serine protease inhibitor, which was confirmed to have an inhibitory effect on CD44 [56, 57]. As shown in Fig. 5f, when the dosage of Bikunin was raised to 1 nM, it showed a significant inhibition on the enhancement of monocytes transmigration rate in Cn infected-HBMEC. Comparing to the control group (17.4 %), the monocytes transmigration rates of Bikunin group was down to 11 and 7.8 %, respectively with dosage 5 nM and 20 nM (Fig. 5f). Similar, Bikunin could also remarkably block enhancement of HIV-1 gp41-I90 in transmigration of monocytes across BBB. Hence, we concluded that, HIV-1 gp41-I90 and Cn enhance the monocyte transmigration across BBB is mediated by CD44.

### HIV-1 gp41-I90 and Cn induce up-regulation of CD44 and ICAM-1 on HBMEC

**Fig. 6:**
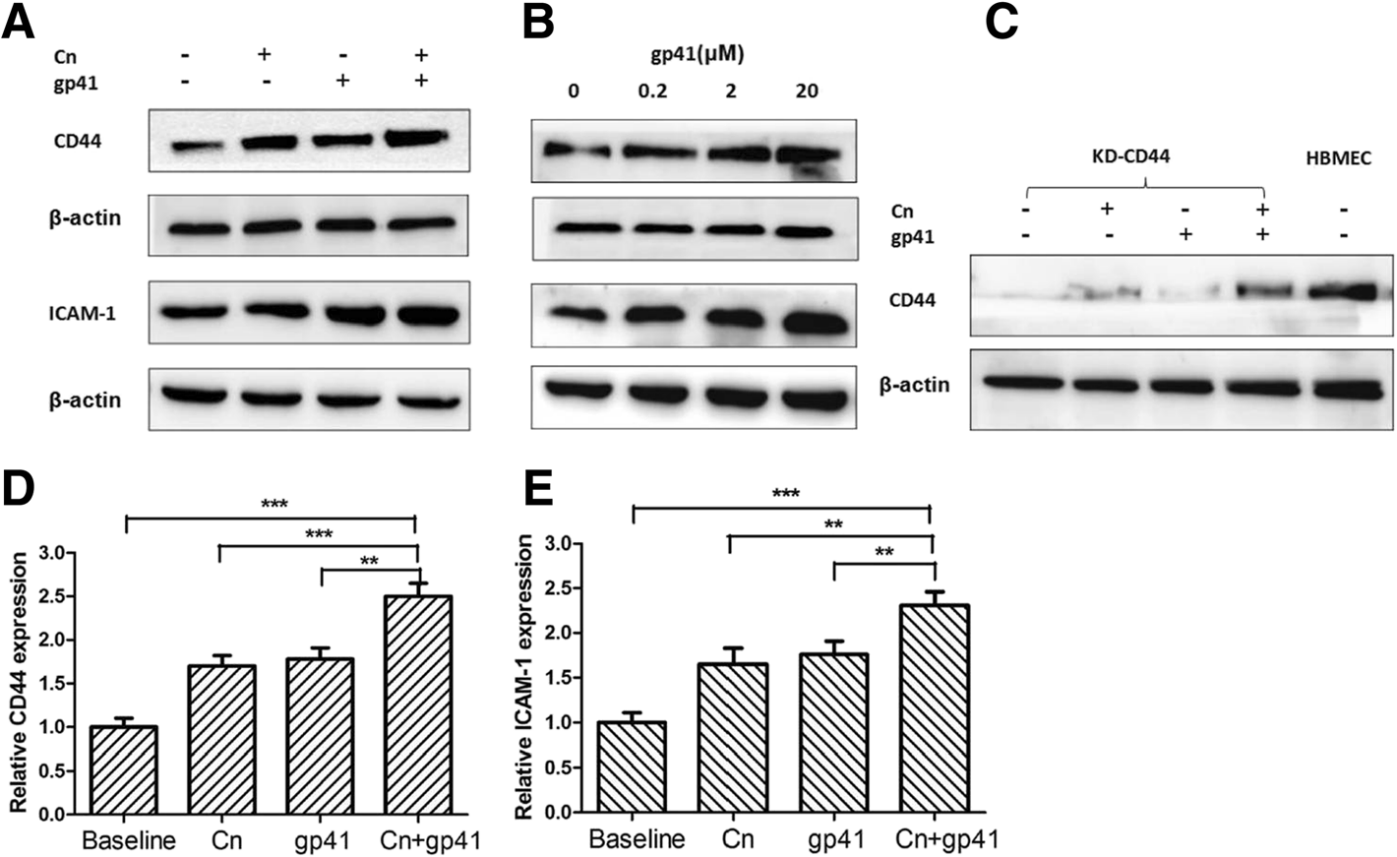
Cn and HIV-1 gp41 enhanced expression of CD44 and ICAM-1 on the HBMEC. Western blotting analyses were performed to measure the up-regulating effect of CD44 (**a**, **c**) and ICAM-1 (**b**) on HBMEC or KD-CD44 HBMEC following exposure to Cn and/or HIV-1 gp41. The β-actin was used as a loading control for each sample. Results showed a significant increase in the CD44/β-actin optical density ratio (*P* < 0.01compared with control). Expression of ICAM-1 (**d**) and CD44 (**e**) was analyzed by BA-ELISAs. Assays were performed in triplicates. Results were expressed as an n-fold increase of protein expression, taking the control as 1. The significant differences between the treatment and control were marked with asterisks (*P < 0.05, **P < 0.01, ***P < 0.001)

After demonstrating the effect of Cn and HIV-1 gp41 in monocytes transmigration across BBB in vitro, we focused on the role of Cn and HIV-1 gp41 in up-regulation of endothelial adhesion molecules that might be involved in monocytes transmigration. Surface expression of endothelial adhesion molecules was studied by using two approaches, western blotting and whole-cell BA-ELISA. As shown in Fig. 6a, b, following 6 h exposure of HBMEC to Cn and HIV-1 gp41-I90, significantly increase in surface expression of ICAM-1 and CD44 was observed. The data of BA-ELISAs were similar to Western blotting, as shown in Fig. 6d, e, the highest expression level of ICAM-1 and CD44 were observed in HBMEC treated with Cn in combination with HIV-1 gp41-I90. Interestingly, as shown in Fig. 6c, expression of CD44 in KD-CD44 HBMEC was up-regulated significantly following co-exposure to Cn and HIV-1 gp41-I90, although there was a slight increase in CD44 upon treatment of Cn or HIV-1 gp41-I90 alone.

### The threshold of induced monocytes transmigration and up-regulated CD44 expression by HIV-1 gp41

**Fig. 7:**
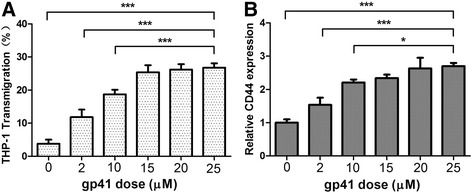
There is a limitation in the induced transmigration of monocytes and up-regulated expression of CD44 by HIV-1 gp41-190. HBMEC were treated with different dose of HIV-1 gp41-I90 (2–25 μM) for 6 h. Transmigration assays (**a**) and BA-ELISAs (**b**) were performed to assess whether transmigration of monocytes and expression of CD44 induced by HIV-1 gp41-190 is dose-dependent. The values represent the mean percent transmigrating THP-1 of triplicate samples and are representative of one experiment from three independent experiments showing similar data. (*P < 0.05, **P <0.01, ***P < 0.001)

In the process of studying the effect of HIV-1 gp41-I90 on the transmigration of monocytes across the BBB in vitro, we were able to observe there is a limitation in the induced monocytes transmigration across BBB by HIV-1 gp41-I90. As shown in Fig. 7a, there was very little increase in transmigration rate 26.24 to 26.81 %, when the concentration of HIV-1 gp41-I90 was increased from 20 to 25 μM. This indicates a saturation level of monocytes transmigration, when the HIV-1 gp41-I90 concentration is approaching 25 μM. We further performed a BA-ELISA to confirm the biological relevance of this finding. HBMECs were treated with different dose of HIV-1 gp41-I90 (from 2 to 25 μM), the whole-cell BA-ELISA were performed as described Methods section to assess the expression of CD44 on the surface of HBMEC. As we expected, there is also a limitation of CD44 expression when the concentration of HIV-1 gp41-I90 was increased from 20 to 25 μM (Fig. 7b). Taken together, the results clearly demonstrate that there is a threshold in the enhancement of monocytes transmigration and over-expression of CD44 induced by HIV-1 gp41-190.

### Redistribution of CD44 to membrane rafts of HBMEC during Cn and HIV-1 gp41-I90 exposure

**Fig. 8:**
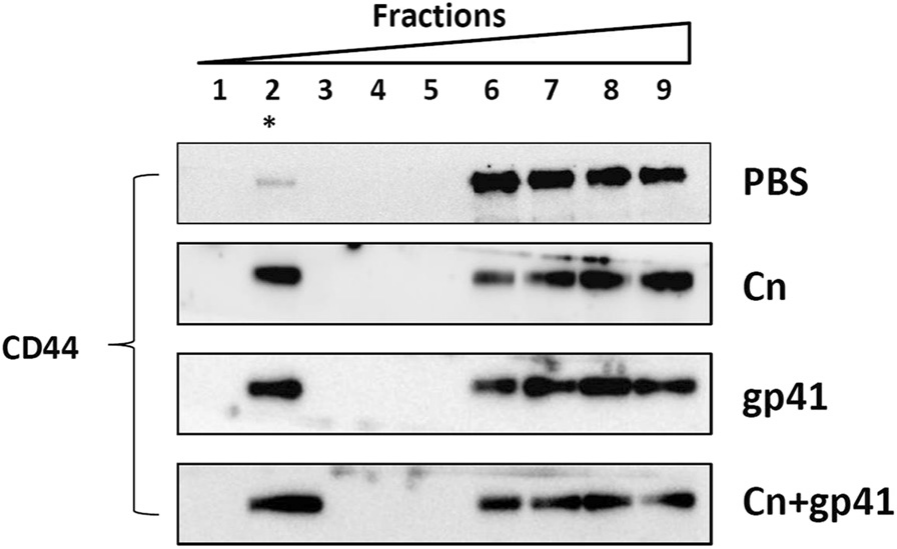
Redistribution of CD44 to membrane rafts during HBMEC exposure to Cn and/or HIV-1 gp41-190. HBMECs were treated with either PBS or Cn, or HIV gp41-I90 or both of them for 6 h. The cells were then lysed in buffer containing 1 % Triton X-100 on ice. Fractionation was performed in OptiPrep^TM^ gradients, and nine fractions were collected. The lipid raft fractions are indicated by * in fraction 2. An equal volume of each sample was analysed by dot blots using antibodies against CD44. Redistribution of CD44 on the membrane rafts was observed in Cn and/or HIV-1 gp41-I90 treated HBMECs

Adhesion molecules recruited to specialized microdomains of lipid rafts is important to regulate intracellular signaling and leukocyte transendothelial migration [58]. Thus, we tested whether Cn and/or gp41-190 could induce CD44 redistribution to the membrane lipid rafts of HBMEC. As CD44 could be a membrane receptor on HBMEC, we used density gradient centrifugation to fractionate membrane rafts. HBMECs treated by Cn and/or HIV-1 gp-41 were lysed in a buffer containing 1 % Triton X-100. The fractionation was performed in OptiPrep^TM^ density gradient centrifugation. After centrifugation, detergent-insoluble membrane lipid raft fractions floated to the interphase between 0 % and 20 % OptiPrep^TM^ layers, peaking at fraction 2 in our study (asterisk in Fig. 8). The loading buffer floated to the top (fraction 1), but soluble proteins or cytoskeleton associated, detergent-insoluble proteins remained in the bottom fractions of the gradient (fractions 3–9). Protein blotting of each fraction was used to examine the distribution of protein components from HBMEC extracts of the Cn and/or gp41-treated samples. As shown in Fig. 8, in untreated HBMEC, CD44 was primarily associated with soluble fractions (fractions 6–9). For the cells treated with either Cn and/or HIV-1 gp41-I90, a significant portion of CD44 had apparently relocated to the membrane rafts as observed in the fraction 2. The result suggested that there was a reorganization of membrane rafts taking place during exposure to Cn and/or HIV-1 gp41-I90, and CD44 became enriched in these membrane rafts on the surface of HBMEC, which facilitates the monocytes transmigrate across the BBB.

### HIV-1 gp41-I90 increased Cn-induced monocyte transmigration, the BBB permeability and injury in vivo

**Fig. 9:**
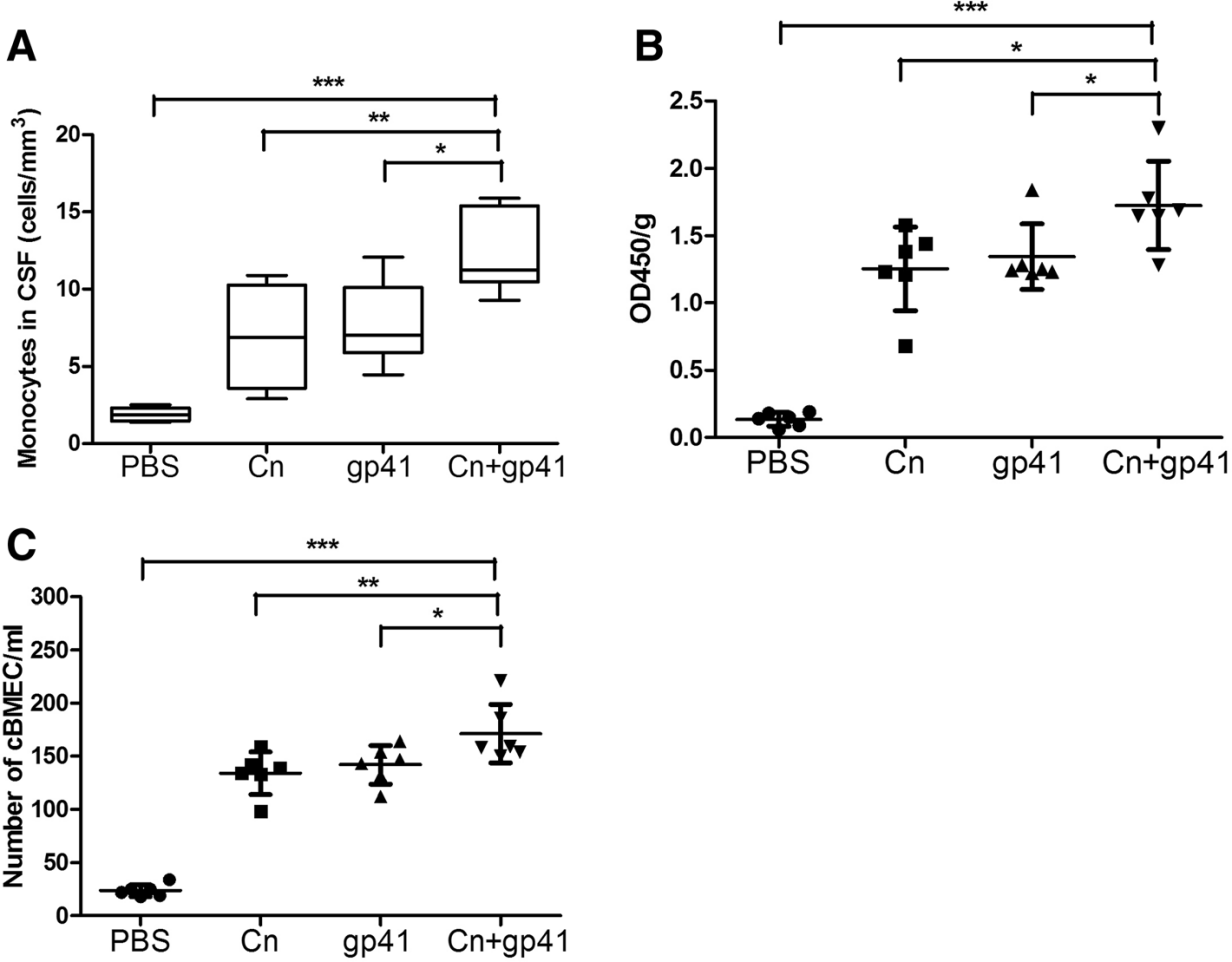
Effects of HIV-1 gp41-I90 on Cn-increased monocyte transmigration, the BBB permeability and injury. **a** CSF concentration of monocytes in mice treated with 10^6^ CFU/ml Cn, 10 μg/g HIV-1 gp41-I90 or both of them. **b** Concentration of EB in brain of mice treated with 10^6^ CFU/ml Cn, 10 μg/g HIV-1 gp41-I90 or both of them. **c** Peripheral blood concentration of cBMEC in mice treated with 10^6^ CFU/ml Cn, 10 μg/g HIV-1 gp41-I90 or both of them. Mice were divided into 4 groups (6 mice/group). Each experiment was performed three times. *P < 0.05, **P < 0.01, ***P < 0.001

To further validate the biological relevance of the in vitro assays, the role of HIV-1 gp41-I90 in the monocyte transmigration across the BBB induced by Cn was tested in the mouse model, as described in Methods section. Animals of the same age were injected with Cn (10^6^ cells) or HIV-1 gp41-I90 (10 μg/g mouse weight) alone or Cn in combination with HIV-1 gp41-I90. Three indexes, monocytes transmigration, EB concentration in brain tissue and number of cBMEC in blood were used to evaluate the pathogenicities of CM. As shown in Fig. 9a–c, all above indexes shown highest mean in the mice injected with Cn in combination with HIV-1 gp41-I90. These results show that HIV-1 gp41-I90 and Cn could synergistically facilitate the monocyte transmigration, increase the BBB permeability (increased EB concentration in brain) and injury (increased cBMEC in blood).

**Fig. 10:**
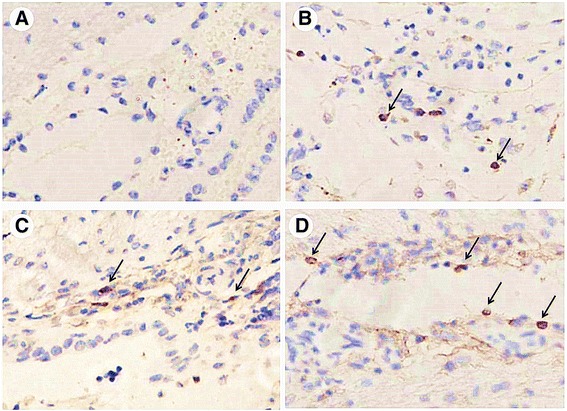
Recruitment of monocytes into the brain parenchyma of mice treated with Cn and/or HIV-1 gp41-I90. C57BL/6 mice were intravenously injected with Cn and/or HIV-1 gp41-I90 via the tail vein. After 24 h injection, mice were anaesthetized with ketamine and lidocaine, and the brains were removed and fixed in 4 % neutral buffered formalin. Immunohistochemistry analysis was performed as described as Methods section. **a** Normal brain. **b** Brian of mice infected with Cn. **c** Brian of mice treated with HIV-1 gp41-I90. **d** Brian of mice treated with Cn + HIV-1 gp41-I90. Arrows indicate infiltrating monocytes. Images are 400 ×

Since most monocytes are recruited into brain parenchyma adjacent to blood vessels during the cryptococcal meningitis, next, we examined the effect of Cn and/or HIV-1 gp41-I90 on recruitment of monocytes into the brain parenchyma of mice. C57BL/6 mice were intravenously injected with Cn and/or HIV-1 gp41-I90 via the tail vein. After 24 h injection, mice were anaesthetized with ketamine and lidocaine, and the brains were removed and fixed in 4 % neutralbuffered formalin. Immunohistochemistry analysis was performed as described as Methods section. As shown in Fig. 10, expose to Cn and/or HIV-1 gp41-I90 was able to significantly increase monocytes transmigration across the BBB. These data suggested that expose to HIV-1 gp41-I90 increased Cn-induced monocytes recruitment into CNS.

## Discussion

Cn is an opportunistic pathogen, which causes fatal meningoencephalitis, especially in AIDS patients. In order to cause meningoencephalitis, Cn must cross the BBB. A great deal of evidence supports the existence of the Trojan horse model of BBB transmigration of Cn. (1) Cn can survive in phagocytic cells via active phagosomal extrusion and spread to the phagocytes [59, 60]; (2) The incidence rate of fungemia and meningoencephalitis is higher in HIV-1-infected patients than that in HIV-1-negative patients because HIV-1 can cause severe monocyte dysfunction in host [61–63]; (3) Cn was carried and transported by circulating phagocytes in the murine model of cryptococcosis in a previous study by Chrétien F. et al. [64]. (4) Cn is a facultative intracellular pathogen and has been shown to survive and multiply inside phagocytes in vitro [65]. Previous research had shown that HIV-1 infection is able to increase the monocyte capacity to migrate across the BBB [36]. In present study, we have suggested that Cn and/or HIV-1 gp41-I90 is able to enhance the transmigration activities of monocytes across BBB by using the in vitro and in vivo BBB models [66]. Importantly, we found that HIV-1 gp41-I90 was able to synergistically enhance the transmigration activity of monocytes in HBMEC infected with Cn and in mice with Cn-caused meningoencephalitis. Thus, we have firstly demonstrated the relationship between HIV-1, Cn and monocytes, which point out a new potential mechanism of invasion for this pathogenic fungus into the brain tissues of HIV-1-infected patients.

Initially, we demonstrated that the transmigration of monocytes across the BBB in vitro could besynergistically enhanced by HIV-1 gp41 protein and Cn. The specificity of the synergistic effect is further confirmed by transmigration assays. Two experiments were designed. In the first experiment, we used H-Cn to examine whether H-Cn and HIV-1 gp41 could synergistically enhance the transmigrate ability of monocytes. Our results have shown that there is no synergistic effect on the transmigration of monocytes with a combination of H-Cn and gp41. Interestingly, we found that H-Cn could also increase monocyte transmigration ability. In the second experiment, HIV Tat and p24 proteins were used. HIV Tat is a regulatory protein that enhances viral transcription and replication, which plays a multifaceted role in pathogenesis of HIV infection, including favouring viral infection, contributing to inflammatory responses and inducing monocyte invasion into the brain [67–70]. Notwithstanding, we found there is no synergistic effect on enhancement of monocyte transmigration upon treatment by a combination of Cn and HIV-1 Tat protein. Similarly, HIV p24, which is a component of the HIV particle capsid, also has no synergistic effect on Cn-mediated enhancement of monocyte transmigration. Taken together, these results suggest that the synergistic enhancement by the HIV-1 gp41 protein on monocyte transmigration across the Cn-infected BBB is viral factor-dependent. This is most likely due to the fact that both HIV-1 gp41 and Cn may elicit a similar signal, such as up-regulating CD44 and ICAM-1 expression (Fig. 6), activating membrane lipid rafts (Fig. 8) and NF-κB [44], to facilitate the transmigration of monocytes. Thus, we speculate that the ectodomain of HIV-1 gp41 may play a role as a trans-predilection factor for cryptococcal CNS invasion, suggesting that the HIV-1 fusion inhibitors targeting gp41, such as T20 and C34, may be helpful in the prevention and treatment of cryptococcal meningitis in HIV/AIDS patients.

CD44 is a well-known type I transmembrane glycoprotein and functions as the major hyaluronan receptor, which is widely distributed in a variety of endothelial cells, mesenchymal cells, hematopoietic stem cells and mesodermal cells and tissues. Although, alternative splicing can produce a large number of different isoforms, they all retain the hyaluronan-binding link-homology region and a common transmembrane and cytoplasmic domain [19]. Recent studies have demonstrated that, the gene that encodes capsule hyaluronic acid synthase is a key virulence gene of Cn. The transmigration process of Cn across the BBB rely on HA binding to the BMEC receptor CD44, which activates the host signal pathway to induce cytoskeleton rearrangement required for Cn invasion [71, 72]. In present study, we used the CRISPR-Cas9 system and CD44 inhibitor to examine whether the enhancement of Cn and HIV-1 gp41-I90 in transmigration of monocytes across the BBB is related to CD44. Indeed, our results revealed that CD44 was involved in the enhancement of monocyte transmigration across the BBB by Cn and HIV-1 gp41.

Beside the effect of inducing monocyte transmigration across the BBB in vitro, in present study, we also found that Cn and/or HIV-1 gp41 could enhance CD44 redistribution to the membrane lipid rafts and up-regulate the expression level of ICAM-1 and CD44, which are two major endothelial adhesion molecules long known for its importance in facilitating leukocyte transmigration. These findings indicate that Cn and HIV-1 gp41-induced migration of monocytes across BMEC in a coordinate manner with up-regulation of ICAM-1 and CD44. Hence, we derived the conclusion that, HBMEC co-exposed with Cn and HIV-1 gp41 exhibited re-distribution of CD44 and over-expression of CD44 and ICAM-1, which lead to enhancement of the adhesion and transmigration rates of monocytes and facilitate cerebral invasion of Cn.

During the process of studying the effect of HIV-1 gp41-I90 on the transmigration of monocytes across the BBB, we found the facilitation of HIV-1 gp41-I90 induced transmigration of monocytes is dose-dependent. When the concentration of HIV-1 gp41 was raised to a certain level, the facilitation get subdued, which remind us that, there is a threshold in the over-expression of CD44 induced by HIV-1 gp41-I90. In order to test the above assumption, different doses of HIV-1 gp41 (2–25 μM) was added to the HBMEC monolayers to observe the transmigration activities of monocyte. These results showed that the facilitation induced by HIV-1 gp41-I90 was significantly saturated with the higher concentrations of the recombinant protein (Fig. 7a). Furthermore, we performed BA-ELISAs to examine whether the over-expression of CD44 induced by HIV-1 gp41-190 is also dose-dependent. As we expected, the expression level of CD44 on HBMEC could became saturated when the concentration of HIV-1 gp41-I90 was increased from 20–25 μM (Fig. 7b). These results have profound clinical significance in antiretroviral therapies for HIV-associated Cryptococoal meningoencephalitis, as it suggests that adherence to antiretroviral therapies may minimize the risk of Cryptococoal neurologic disease.

## Conclusions

In conclusion, HIV-1 gp41-I90 and Cn is able to promote the adhesion and transmigration activities of monocyte, and the co-exposure of HIV-1 gp41-I90 and Cn further accelerate the adhesion and transmigration activities of monocyte. This may result in a deteriorating cryptococcosis in the infected host. The details for how the HIV-1 enhances cryptococcal invasion into the human brain remain unclear. However, our studies provide the enlightenments to establish the exact mechanism of inflammatory responses induced by the HIV-1 gp41-I90 ectodomain often co-morbid with Cn that lead to HIV-1-associated CM, and provide a theoretical basis for new ways to effectively combat opportunistic infections of the central nervous system in AIDS patients.
